# Next generation sequencing technologies for a successful diagnosis in a cold case of Leigh syndrome

**DOI:** 10.1186/s12883-018-1103-7

**Published:** 2018-07-20

**Authors:** Paolo Aretini, Chiara Maria Mazzanti, Marco La Ferla, Sara Franceschi, Francesca Lessi, Veronica De Gregorio, Claudia Nesti, Angelo Valetto, Veronica Bertini, Benedetta Toschi, Roberta Battini, Maria Adelaide Caligo

**Affiliations:** 1 Fondazione Pisana per la Scienza ONLUS, Via Ferruccio Giovannini, 13, 56017 San Giuliano Terme, Pisa Italy; 2 Molecular Medicine, IRCCS Fondazione Stella Maris, Viale del Tirreno 331, 56128 Calambrone, Pisa Italy; 3 Cytogenetics Laboratory, Santa Chiara University Hospital, Via Roma 67, 56126 Pisa, Italy; 4 Department of Clinical and Experimental Medicine, Santa Chiara University Hospital, Via Roma 67, 56126 Pisa, Italy; 5 Department of Clinical and Experimental Medicine, University of Pisa , Via Savi P, 56126 Pisa, Italy; 6 Molecular Genetics Laboratory, Santa Chiara University Hospital, Via Roma 67, 56126 Pisa, Italy; 7 Department of Developmental Neuroscience, IRCCS Fondazione Stella Maris, Viale del Tirreno 331, 56128 Calambrone, Pisa Italy

**Keywords:** Leigh disease, ECHS1 gene, Exome analysis

## Abstract

**Background:**

Leigh Syndrome (LS, OMIM 256000) is an early-onset, progressive neurodegenerative disorder characterized by broad clinical and genetic heterogeneity; it is the most frequent disorder of mitochondrial energy production in children. LS inheritance is complex because patients may present mutations in mitochondrial DNA (mtDNA) or in nuclear genes, which predominantly encode proteins involved in respiratory chain structure and assembly or in coenzyme Q10 biogenesis. However, during the last 15 years, the discovery of several genetic mutations and improved knowledge of the natural history of LS has significantly increased our understanding of this mitochondrial disorder.

**Case presentation:**

Here we describe a 19-year-old male with clinical and neuroimaging LS diagnosed at 3 years of age. Genetic analyses of the whole mtDNA for maternally inherited LS (MILS) and neuropathy ataxia retinitis pigmentosa (NARP) syndrome failed to reveal any pathogenic mutations.

**Conclusions:**

Recently, a missense mutation in *ECHS1* and a ~ 35 kb deletion in 10q26.3 involving the region including the gene were identified by WES (whole exome sequencing), uncovering the genetic diagnosis clinically hypothesized for 15 years. We also report the long-term follow-up of this patient, showing a comparison with classical LS or other Leigh-like pictures.

**Electronic supplementary material:**

The online version of this article (10.1186/s12883-018-1103-7) contains supplementary material, which is available to authorized users.

## Background

Leigh Syndrome (LS, OMIM 256000) is a rare, heterogeneous, progressive neurodegenerative disorder caused by mutations in mitochondrial DNA (mtDNA) or in nuclear genes, usually presenting in infancy or early childhood [1, 2]. Atypical or later onset cases have been reported in the literature and are referred to as Leigh-like diseases [3]. The clinical presentation is variable and includes psychomotor delay or regression, acute neurological or acidotic episodes, hypotonia, ataxia, spasticity, movement disorders, and corresponding anomalies of the basal ganglia and brain stem on magnetic resonance imaging (MRI) [3–5]. The prognosis is generally poor with rapid deterioration of cognitive and motor function resulting in death within months or years [6, 7]. Since the identification of the first pathogenic mutation in a LS patient in 1991, more than 75 disease genes have been identified, most of them thanks to the introduction of next-generation sequencing (NGS) technology. Recently, enzymes of the valine degradation pathway have also been shown to cause LS [8, 9]; in particular, mutations in enoyl-CoA hydratase (*ECHS1*), a nuclear gene encoding a mitochondrial matrix enzyme catalyzing the second step of the ß-oxidation spiral of fatty acids, has been associated with LS in several patients [9–11].

Here we describe a 19-year-old man who was diagnosed at 3 years of age with LS using clinical and neuroimaging data. MILS and NARP syndromes due to mtDNA mutations were excluded. We confirmed the clinical diagnosis hypothesized for 15 years by using whole exome sequencing (WES) analysis, which identified a missense mutation in *ECHS1,* and by array CGH analysis, which evidenced the deletion of the entire gene. In addition, we report the long-term follow-up of this patient compared with other classical LS or Leigh-like pictures.

## Case presentation

**Fig. 1:**
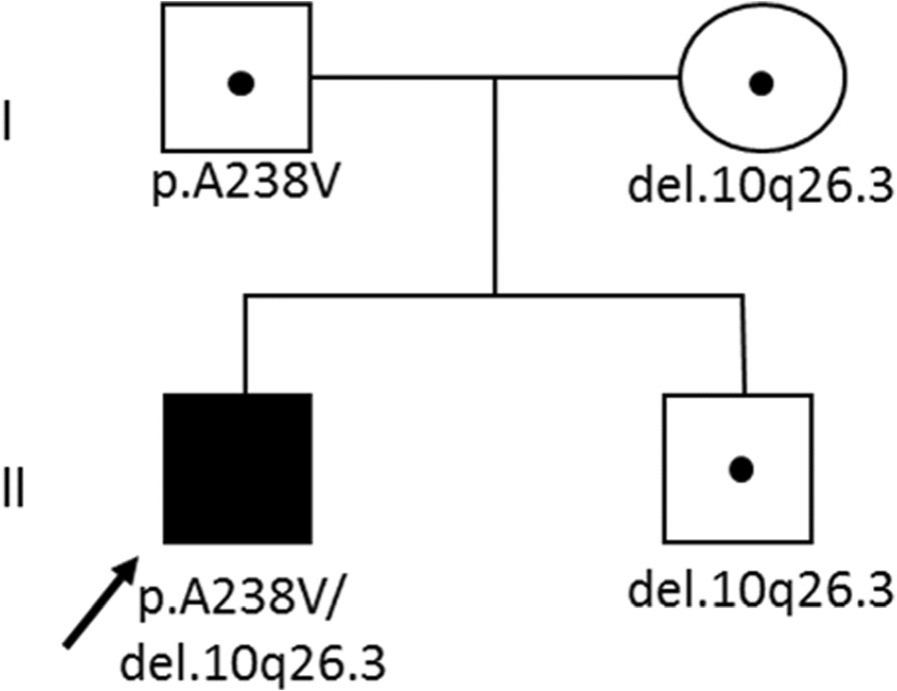
Pedigree of the family. Black symbol represent the proband; healthy family members are shown with open symbols. Small dot: *ECHS1* mutation carriers

The proband is a 19-year-old male born from non-consanguineous parents of Caucasian origin, after a normal pregnancy at 40 weeks of gestation with normal birth measurements (weight 4150 kg, length 52 cm, and cranial circumference 36 cm). Both parents and the 18- year-old brother are healthy (Fig. 1).

Nystagmus, convergent strabismus, and mild lower spasticity appeared at 7 months of age as the first symptoms of the disease, and subsequently, developmental psychomotor regression was observed. In particular, the child at 14 months lost the ability to walk alone, presenting ataxic signs, and at 16 months chorea of the arms and dystonia of the trunk appeared.

**Fig. 2:**
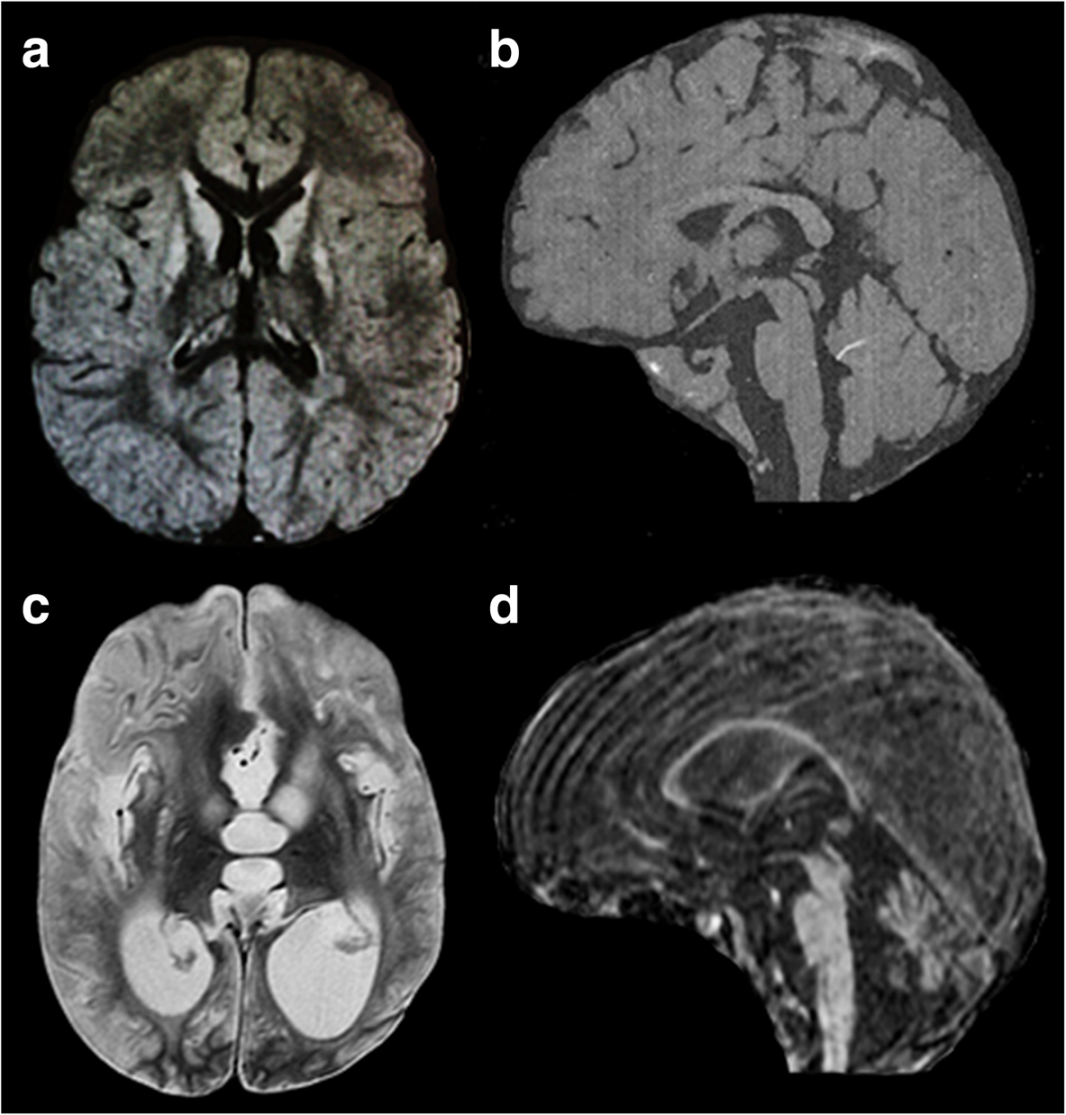
1.5 T low-quality images of the first MRI examination of the boy (16 months); the best available images were chosen. Axial FLAIR (**a**) slice shows the typical pattern of LS with basal ganglia involvement (caudate and lenticular nuclei) and mild involvement of thalami bilaterally. Sagittal T1 slice (**b**) shows normal tropism of the corpus callosum and cerebellum. Last MRI examination at 12 yrs. (**c**-**d**) shows marked progression of basal ganglia involvement with enlargement of lateral and third ventricles, indicating global atrophy. Sagittal FLAIR slice (**d**) highlights marked thinning of the corpus callosum, a consequence of brain atrophy, and severe cerebellum atrophy. The presence of significant movement artifacts is related to MRI without anesthesia, because of critical conditions of the patient

We performed the first MRI showing the typical pattern of Leigh Syndrome with the presence of bilaterally hyperintense signals in the basal ganglia and thalami and of periventricular white matter (Fig. 2a-b); proton magnetic resonance spectroscopy (1H MRS) showed a lactate peak at 1.33 ppm.

The metabolic pattern was characterized by increased levels of plasma lactate, alanine, and valine and reduced levels of citrulline; an increase in 3 methylglutaconic acid was observed in urinary organic acids. Electroencephalogram (EEG) did not show epileptic discharges. A muscle biopsy performed at 18 months showed signs of a mild myopathic process with non-specific abnormalities of oxidative reactions. Respiratory chain activities revealed a slight reduction of complexes II and III.

At 26 months the child presented a dyskinetic tetraparesis associated with hyposthenia of the trunk and limbs. No cardiac, endocrine, gastrointestinal nor renal involvement was observed. The Griffiths scale, which examines the cognitive profile, showed a moderate intellectual disability. The clinical condition remained stable up to 9 years of life, when he presented drug resistance and generalized tonic clonic and myoclonic seizures. Spastic tetraparesis worsened and neurological changes, such as dysarthria/dysphagia, loss of eye contact, and axial and limb dystonia were occurring during acute viral infections. Cognitive deterioration was progressively evident in association with a regression of language skills.

In order to treat the severe spasticity and associated dystonia, an intratecal baclofen pump was implanted, and at 10 years the child was continuously fed by parenteral gastrostomy (PEG). During this period, he also presented constant rhythmic jerky movements of the right arm and of the soft palate, which were indicative of palatal myoclonus status. EEG recording over time showed recurrent focal seizures clinically associated with motor manifestations and during palatal myoclonus, suggesting an ongoing non-convulsive status epilepticus, treated with carbamazepine and benzodiazepine in combination with phenytoin, already administered. No adverse effects were observed, and remission of the seizures was completely obtained; subsequently, phenytoin was gradually reduced and stopped.

At 11 years and 8 months of age, the spastic tetraparesis associated with dystonic movement disorders was complicated by severe scoliosis. The boy also presented a severe intellectual disability, an epileptic encephalopathy, and cerebral visual impairment. Bilateral optic atrophy and sensorineuronal deafness were discovered at 13 years: flash visual evoked potential was not identifiable, whereas brainstem acoustic evoked potential was altered with pons and mesencephalon wave involvement. At around 12 years of age, a second MRI was performed, which showed marked progression of basal ganglia involvement with enlargement of lateral and third ventricles, evident signs of global cerebral atrophy (Fig. 2c-d). A mild double peak of lactate was evident on 1H MRS. A new analysis of muscle tissue and oxidative-phosphorylation enzymes was performed at the age of 13 years, and despite the severe clinical picture, only mild changes in the reduction of complexes II and III were confirmed. Over the course of the disease, limb spasticity with stable elbow and knee contractures were evident, but dystonic movements diminished, and no spontaneous movements were visible. Cardiac and renal function remained unchanged. Constipation was observed.

A tracheostomy was performed at 17 years when he had a bronchopneumonic infection, and his respiratory condition worsened. At 19 years, the patient showed a persistent vegetative state with partial symptomatic drug-resistant epilepsy, pendular nystagmus as seen in blind patients, spastic tetraparesis with hip dislocation, valgus pronatus deformation of the feet, severe scoliosis, and stable flexion of the right arms.

WES analysis was performed on the proband and the asymptomatic father’s and mother’s DNA. After filtering criteria (described in detail in Additional file 1) were applied, only four missense mutations were left. None of the missense variants were exclusive of the affected individual (Table 1) except one in the *ECHS1* gene: the c.713C > T/p.Ala238Val mutation was present in the proband in an apparent homozygous state, whereas the father was found to be heterozygous. This mutation, present in the ExAC Browser (http://exac.broadinstitute.org/; MAF = 0.000016), is predictably damaging when examined in silico using Polyphen2 and has already been reported in the compound heterozygous form in a family presenting with LS [3].

**Table 1:** Presence of Leigh-related missense mutations in samples

Genes	Designation	Proband	Mother	Father
PNPT1	c.1519G > T; p.Ala507Ser	Het	–	Het
SYNE1	c.23315G > A; p.Arg7772Gln	Het	Het	–
SYNE1	c.13909G > A; p.Asp4637Asn	Het	Het	–
ECHS1	c.713C > T; p.Ala238Val	Homo	–	Het

**Fig. 3:**
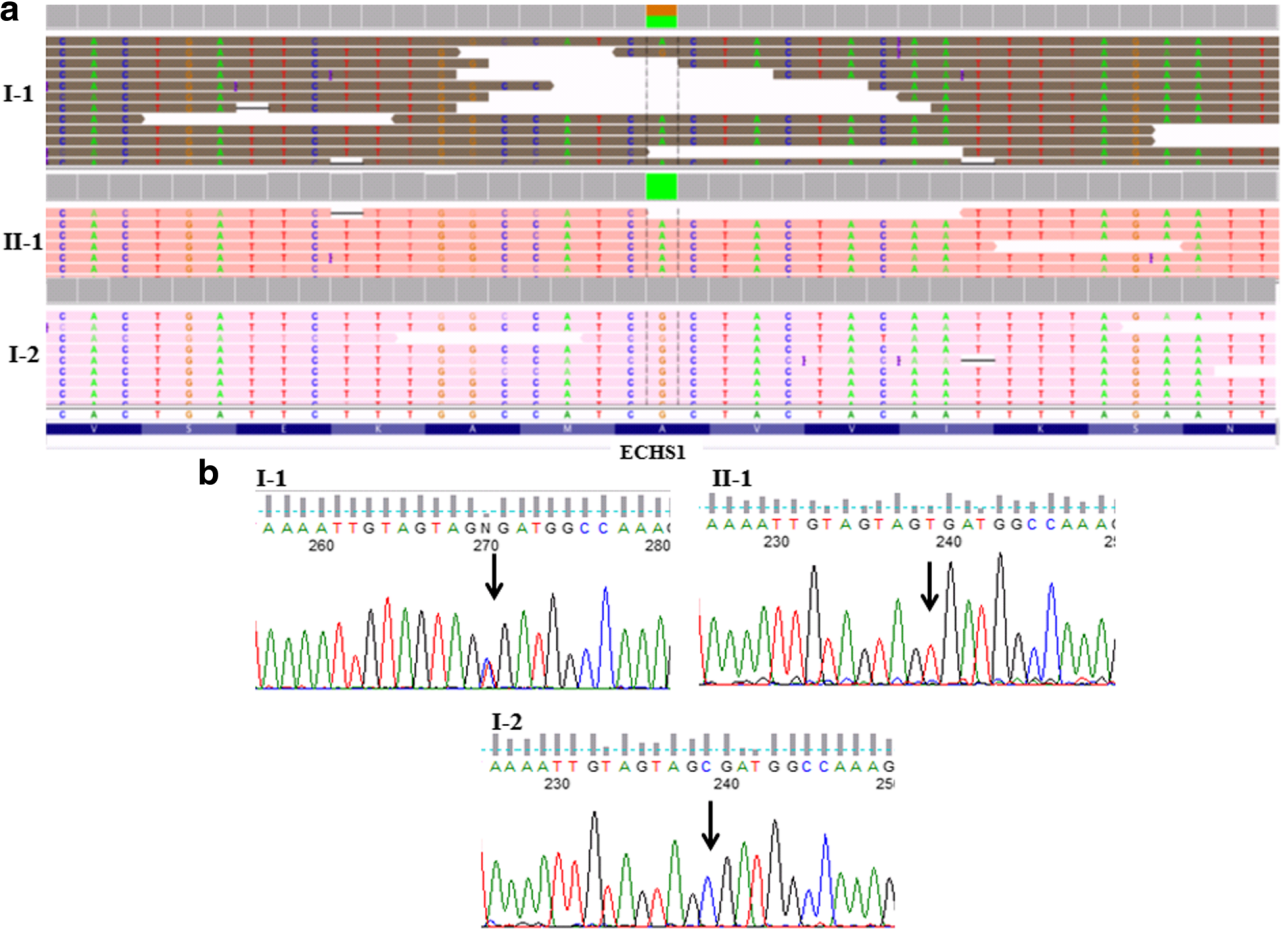
IGV visualization (**a**) and sequence chromatographs (**b**) of the *ECHS1* region flanking the missense mutation c.713C > T/p.Ala238Val from the affected patient (II-1, indicated by an arrow), his father (I-1) and mother (I-2). In IGV the green color corresponds to the nucleotide base A; the brown to the nucleotide base G

Manual inspection of bam files by Integrative Genome Viewer (IGV) as well as Sanger sequencing of the flanking region revealed the lack of this mutation in the mother (Fig. 3a and b). To further investigate the chromosomal region containing *ECHS1*, we used CeQer, a software program able to detect copy number variation from exome data. We detected a deletion in an extended region of chromosome 10 (from 135,120,573 to 135,187,238) involving five genes: *ZNF511*, *CALY*, *PRAP1*, *FUOM,* and *ECHS1*. This deletion was present in the proband and in his mother but not in the father (Additional file 1: Figure S1).

**Fig. 4:**
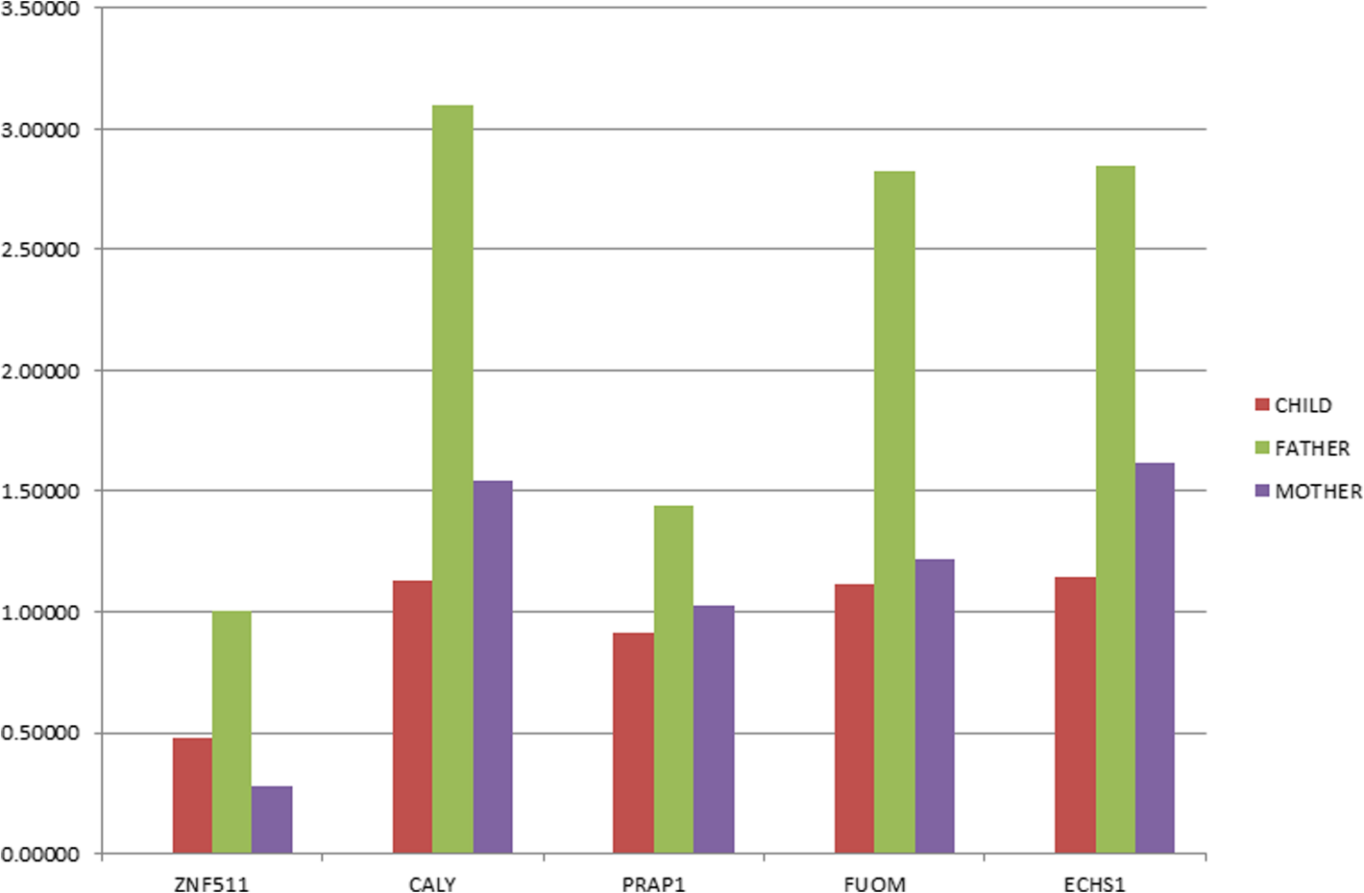
RT-PCR shows gene profiles on genomic DNA. To determine relative gene load, a ∆∆Cq method was performed from qPCR data with GAPDH as an endogenous reference gene. These data show a depletion of the *ZNF511*, *CALY*, *PRAP1*, *FUOM,* and *ECHS1* genes in two cases (red and purple histograms) with respect to the father (green histogram), who does not present a deletion of the region of interest

The array CGH identified in the proband a microdeletion of at least 35 kb in 10q26.3 (Additional file 1: Figure S2). This deletion was also detected in the mother. The maternal grandmother was negative; therefore, it is likely that this deletion was inherited from the grandfather (sample not available). In order to confirm whether the deletion led to a loss of copy number of the five included genes, a real-time PCR assay was performed. The results showed that the patient and his mother had a decreased copy number in all genes compared with that of the father (Fig. 4). The Additional file 1 describes all the methods used in this paper and the additional file figures not shown in the text.

## Discussion

In this study, we report the case of a 19-year-old man with a diagnosis of Leigh Syndrome. By WES analysis, a missense mutation in the nuclear gene *ECHS1* was identified in the proband and his father in the heterozygous state and a deletion in 10q26.3 involving the region including *ECHS1* was found in the proband and his mother. CGH analysis and RT-PCR confirmed this deletion.

In the recent years, WES technology has been extensively used for clinical studies; since a significant part of mutations exists in the exons, this approach has achieved great results for identifying Mendelian disease genes. Success of exome sequencing in revealing pathogenic mutations and identifying genes have been demonstrated by several studies [12, 13].

Using WES, Haack et al. [10] identified 10 unrelated individuals carrying compound heterozygous or homozygous mutations in *ECHS1* and exhibiting very heterogeneous phenotypes. Recently, two siblings were described with fatal Leigh disease who had increased excretion of several metabolites that are features of 3-hydroxyisobutyryl-CoA hydrolase (HIBCH) deficiency [9]. Further papers described *ECHS1* mutations variously associated with vacuolating leukoencephalopathy, basal ganglia abnormalities or a slow neurodegenerative condition with global brain atrophy or a single episode of metabolic acidosis. All patients presented with lactic acidosis [3, 13, 14].

Our patient presented a clear clinical overlap with the reported cases with *ECHS1* or *HIBCH* deficiency, and the same mutation (p.Ala238Val) was found in two patients [3]. This variant, together with other four missense mutations, is present in the large enoyl-CoA hydratase/isomerase domain, responsible for substrate binding and the catalytic activity of the enzyme and is predicted by the MuPro and Auto-Mute software programs to decrease protein stability [15, 16]. *ECHS1* is a nuclear gene involved in several metabolic pathways involving fatty acids and amino acids. The substantial discrepancy between an expected moderate impairment of mitochondrial fatty acid oxidation and the severe clinical presentation of ECHS1-deficient individuals may account for additional pathogenic mechanisms. In addition, ECHS1 has been suggested to be involved in L-isoleucine, L-valine, and L-lysine oxidation using tiglyl-CoA, 2-methacrylyl-CoA, or crotonyl-CoA, respectively, as a substrate. The mitochondrial respiratory chain does not appear to be severely impaired in *ECHS1* patients, as demonstrated also in our case. Possible biomarkers of the disease are not currently available; the methacrylate metabolites and/or 2-methyl-2,3-dihydroxybutyric acid could represent an important hint. In our patient, an increase of plasma levels of alanine and valine and the presence of 3-methylglutaconic acid in urine suggested an inherited disorder of mitochondrial energy metabolism [10]. Our patient presented with many clinical characteristics associated with LS, including general (failure to thrive), neurologic (hypotonia, dystonia, developmental delay, MRI findings), audiologic (sensorineural hearing loss) and ophthalmologic (nystagmus, optic atrophy) features but, given the age of onset of the disease and the severity of the clinical picture his outcome was unexpected. The young man is still alive and the interruption of epilepsy in recent years may be related to the severe brain atrophy developed over time.

## Conclusions

Despite the advent of NGS technologies, proved to be successful in the identification of disease-causing genes, the exact diagnosis of genetically heterogeneous diseases, such as LS, remains difficult. In our case, the application of complementary genetic methodologies has allowed the molecular diagnosis in a case that would have not be resolved with standard analyses.

The description of additional *ECHS1* patients with their longitudinal follow up, could contribute to a better definition of the clinical spectrum of *ECHS1* deficiency and to reach an early diagnosis, helpful for optimal genetic counselling.

## Additional file

Additional file 1: Methods used to perform genetic analyses. (DOCX 350 kb)

## Abbreviations

1H MRS: Proton magnetic resonance spectroscopy
EEG: Electroencephalogram
IGV: Integrative genome viewer
LS: Leigh syndrome
MILS: Maternally inherited Leigh syndrome
MRI: Magnetic resonance imaging
mtDNA: mitochondrial DNA
NARP: Neuropathy ataxia retinitis pigmentosa
NGS: Next generation sequencing
PEG: Parenteral gastrostomy
WES: Whole exome sequencing
