# Supplementary material for: Next generation sequencing technologies for a successful diagnosis in a cold case of Leigh syndrome
Source: BMC Neurol. 2018 Jul 20;18:99. doi: 10.1186/s12883-018-1103-7 (PMC6054728; doi:10.1186/s12883-018-1103-7)
Supplement: Supplementary file 1 — Methods used to perform genetic analyses. (DOCX 350 kb) [file 12883_2018_1103_MOESM1_ESM.docx]

**METHODS**

Having obtained parental written informed consent, we collected genomic DNA from peripheral blood from the proband and his parents. The patient was followed up and treated at IRCCS Stella Maris, Pisa. This study was approved by the Regional Pediatric Ethics Committee (Meyer Hospital, Florence).

**Exome Analysis**

The library was sequenced to mean 33x coverage on the Life Technologies Ion Proton sequencing platform (Ion Torrent, Life Technologies, Grand Island, NY) for next-generation sequencing. Genomic DNA was extracted from whole blood using standard methods. To sequence our samples, we used an Ion PI Sequencing 200 kit (Ion Torrent, Life Technologies, Grand Island, NY). Sanger sequencing was performed using an ABI 3500 Genetic Analyzer (Life Technologies, CA, USA) to validate identified variants.

**Bioinformatic Analysis**

After the Ion Proton run, data were automatically analyzed by using the Ion Torrent server, previously set for alignment to the human genome (hg19 version). The vcf file, containing SNVs and small indels, was used as the input in Enlis Genome Research (Enlis, LLC, Berkeley, CA), a commercial tool to annotate and filter genetic variants. Read alignments were visualized using IGV (<http://www.broadinstitute.org/igv)>.

After variant call and annotation, 47303 variations were found in the sample of the proband, 15913 of which were protein-coding variations. We used Enlis Genome Research to filter the VCF file. We started by using the following filter: quality score >= 10, read depth >=10, allele frequency (as 1000 Genome Project and Exome Aggregation Consortium) < 1% and protein impact involving missense, non-sense, frameshift, and splice disrupt mutations. For missense mutations we used the Dann Model [1] to select the predicted deleterious alterations. Silent variations that do not result in any change in amino acid in the coding region were excluded. After this filtration step, we found 746 deleterious or predicted deleterious mutations. At this point we further refined the research by filtering the sample using a list of common and less common genes involved in LS or related syndromes ([2] and Online Mendelian Inheritance in Man, OMIM [3]). CEQer software (CS)[4] was used to analyze copy number variations (CNVs) and loss of heterozygosity (LOH). We performed the analysis by using a log2 ratio cut-off of 0.8.

**Array CGH**

Array CGH was performed according to standard protocols. Microarray experiments were performed on DNA microarrays 4x180K (Agilent) that have a 20 kb average probe spatial resolution. Microarrays were scanned with an Agilent scanner G256BA. Hybridization data analysis was carried out with Cytogenomics software. A search for copy number variation regions was performed using the Database of Genomic Variant (http://projects.tcag.ca/variation/).

**Real-time PCR assay**

We performed real-time PCR on DNA to verify the copy number variation in order to validate the presence of the deletion found by NGS analysis and array CGH. We analyzed the five genes encompassed by the deletion, namely ZNF511, CALY, PRAP1, FUOM, and ECHS1.

**Additional Figures**


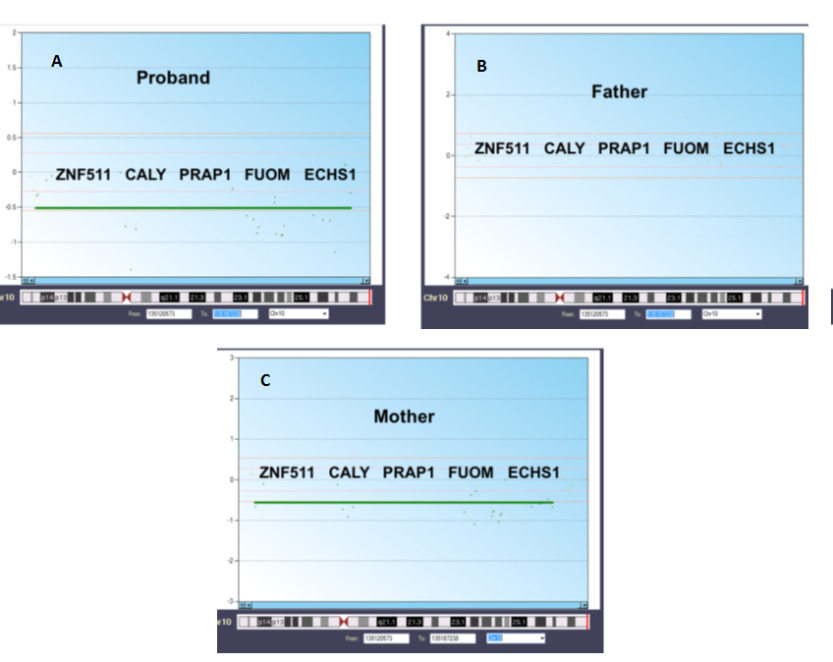


**Figure S1**. Graphic output of CeQer software shows the 10q26.3 deletion (green line). The deletion is present in the proband and mother (A-C) but not in the father (B).


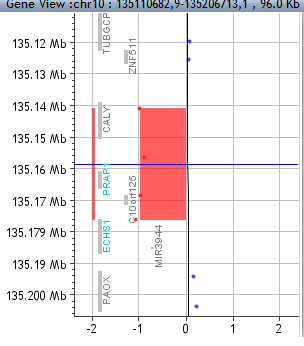


**Figure S2.** Array-CGH showing the 10q26.3 deletion in the proband (highlighted in red).

**References of S1**

1. Quang D, Chen Y, Xie X. DANN: a deep learning approach for annotating the pathogenicity of genetic variants. Bioinformatics. 2015;31:761–3.

2. Lake NJ, Compton AG, Rahman S, Thorburn DR. Leigh Syndrome: One disorder, more than 75 monogenic causes. Ann. Neurol. 2015.

3. Amberger JS, Bocchini CA, Schiettecatte F, Scott AF, Hamosh A. OMIM.org: Online Mendelian Inheritance in Man (OMIM®), an online catalog of human genes and genetic disorders. Nucleic Acids Res. 2015;43:D789–98.

4. Piazza R, Magistroni V, Pirola A, Redaelli S, Spinelli R, Redaelli S, et al. CEQer: a graphical tool for copy number and allelic imbalance detection from whole-exome sequencing data. Veitia RA, editor. PLoS ONE. 2013;8:e74825.
